# Effects of European Bilberry Extract on Skin Aging Associated with Consumption of a High Temperature Roasted Diet in Rats

**DOI:** 10.3390/nu18193195

**Published:** 2026-09-27

**Authors:** Ruijie Cheng, Nanpeng Zhao, Lihui Shen, Yunhong Sun, Ning Yang, Ruikun He, Liegang Liu, Xiaoxing Mo, Hong Yan

**Affiliations:** 1 MOE Key Lab of Environment and Health, Department of Health Toxicology, School of Public Health, Tongji Medical College, Huazhong University of Science and Technology, 13 Hangkong-Road, Wuhan 430030, China; 2 Hubei Key Laboratory of Food Nutrition and Safety, Department of Nutrition and Food Hygiene, School of Public Health, Tongji Medical College, Huazhong University of Science and Technology, 13 Hangkong Road, Wuhan 430030, China; 3 BYHEALTH Institute of Nutrition & Health, Guangzhou 510663, China

**Keywords:** advanced glycation end products, UHPLC-MS/MS, anthocyanin, skin aging, skin barrier

## Abstract

**Background/Objectives:** Skin aging is accompanied by increased protein glycation in the skin, suggesting a potential link between dietary advanced glycation end products (AGEs) and skin aging. European bilberry extract (EBE), rich in anthocyanins, has shown potential in reducing AGEs; however, its protective effects against AGE-induced skin aging and the underlying mechanisms remain unclear. We aimed to investigate the association between roasted high-AGE diet exposure and skin aging, and to evaluate the protective effects of EBE and its potential mechanisms. **Methods:** A long-term roasted high-AGE diet exposure model was established in rats using a roasted high-AGE diet. Rats received EBE (100 mg/kg/day) or vehicle by oral gavage for 16 months. AGE levels were quantified by UHPLC-MS/MS. Skin aging phenotypes were assessed by histomorphometry and senescence-related biomarkers. Lipidomics analysis was performed to evaluate alterations in skin lipid profiles and the effects of EBE. **Results:** In rats, high-AGE feeding increased circulating and skin-deposited AGEs, reduced epidermal and stratum corneum thickness, and elevated senescence markers. Lipidomics revealed significant lipid remodeling, including decreased unsaturated fatty acids and glycerolipids; these lipid alterations were consistent with changes in lipid pathways related to barrier integrity, energy storage, and inflammatory signaling. EBE supplementation reduced systemic and cutaneous AGE accumulation, improved skin structure, attenuated senescence, and partially restored lipid homeostasis, including lipids associated with barrier function, energy storage, and inflammation. **Conclusions:** A roasted high-AGE diet is associated with and may accelerate skin aging, accompanied by lipid metabolism disruption and activation of inflammation-related pathways. EBE alleviates roasted high-AGE diet-induced skin aging and barrier-related molecule disruption, along with restoring lipid homeostasis and suppressing pro-inflammatory responses.

## 1. Introduction

Skin aging is characterized by progressive functional decline and impaired regenerative capacity. Evidence has shown that skin aging may affect biological aging, highlighting the importance of understanding its underlying mechanisms and identifying effective protective strategies [1]. Advanced glycation end products (AGEs), a group of heterogeneous chemicals [2], have been implicated in skin aging. Current studies have revealed the potential of AGEs to promote skin aging. Exposure to individual AGEs in reconstructed skin models has been shown to induce skin-aging-like changes and alter barrier-related molecules [3]. A recent mouse study showed that 16 weeks of oral exposure to a high-AGE diet was associated with skin inflammation and aging-related phenotypes [4]. Collectively, these findings suggest that AGEs may contribute to skin aging and barrier-related alterations. Dietary intake is an important source of AGE exposure in vivo and may increase the systemic AGE burden [5]. Nevertheless, the effects of long-term dietary AGE exposure on the skin, a complex organ system, remain insufficiently understood. In vivo studies examining long-term dietary exposure remain limited.

Lipids are major components of the epidermis and play a key role in energy storage, signal transduction, metabolism, cell development, and membrane structure [6], and the alteration in the aging process has been recognized gradually [7]. Part of the skin lipids located at the epidermis contribute to the chemical barrier; abnormal lipid composition of the stratum corneum culminates in defective barrier function [8]. An in vitro study of glycation exposure indicated upregulated fatty acid synthesis and promoted barrier disruption, probably via the alterations of physiological properties and cytotoxicity of saturated fatty acids [9]. Due to the contribution of inflammatory responses to barrier dysfunction [10], and the indication of lipid metabolism alteration prior to skin glycation [11], lipid disorders and the following inflammation might affect barrier function during skin aging. Since the lipid profile largely depends on dietary intake in current human lifestyles [12], we reasonably believe that dietary AGE exposure may be associated with alterations in skin lipid profiles, which might contribute to the skin aging process and accompanying barrier dysfunction, possibly involving inflammation. Up to now, few studies of the association between dietary AGE exposure and skin lipid profile changes have been reported.

As an extract with antioxidant properties, European bilberry extract (EBE) has also shown potential protective effects on the skin. In vivo, EBE promoted barrier function in the epithelial system [13]. Topical EBE administration and oral supplementation with a blueberry-based combination formulation also showed a protective effect on skin moisture and barrier function [14,15]. In addition, the protective effect on inflammation and inhibition of lipid oxidation have been confirmed [16]. In light of the existing literature, we expected an anti-aging effect of EBE on the skin by oral supplementation through regulating the lipid profile and attenuating inflammation.

In this study, we aimed to investigate the effect of long-term exposure to a roasted, AGE-rich diet on skin aging and alterations in lipid profiles using rats fed with a roasted high-AGE diet, and to evaluate the protective effects of EBE on the lipid profile and skin barrier-related molecular changes. We hypothesized that a roasted high-AGE diet promotes skin aging and impairs barrier-related molecular features potentially in association with lipid alterations, involving inflammatory responses, and that EBE supplementation may attenuate these effects.

## 2. Materials and Methods

Detailed experimental procedures, including reagent and instrument information, are provided in the Supplementary Materials.

### 2.1. Preparation of EBE

BYHEALTH Co., Ltd., Guangzhou, China provided EBE. Fresh bilberries were soaked, and pure extracts were isolated. The extracts were then standardized and dissolved. UHPLC-MS was used to quantify the specific components of this product. The major active constituents of EBE were 36% anthocyanins (including 15 kinds of anthocyanins). Further details of the analytical methods and extract composition were reported in our previous study [17].

### 2.2. Preparation of a High-AGE Diet

A standard chow diet (AIN-93M, Medison Biopharmaceutical Co., Ltd., Jiangsu, China) was roasted at 150 °C for 30 min to obtain a high-AGE diet, as previously described [5]. The four main types of AGEs were significantly increased during processing under this condition. No significant differences were seen in calories and the main nutrients in the roasted diet, as reported in our previous study.

### 2.3. Animals

Twelve-week-old female *Sprague-Dawley* (SD) rats were purchased from Beijing, China HFK Bio-Technology Co., Ltd. and reared in a specific pathogen-free (SPF) facility (22 ± 2 °C, 50% ± 5%, 12 h dark/12 h light cycle) with free access to irradiated food and water. The Institutional Animal Care and Use Committee at Tongji Medical College, Huazhong University of Science and Technology, approved the animal experiments (IACUC number: 4831).

### 2.4. Study Design

After one month of adaptation, 24 rats were randomly divided into three groups using a random number table: the control (CON) group (*n* = 8) was fed with a standard chow diet (AIN-93M), while the AGE group (*n* = 8) and the EBE group (*n* = 8) received a high-AGE diet (roasted diet). Rats in the EBE group were gavaged with European bilberry extract 100 mg/kg/d dissolved in 0.9% normal saline. Additionally, the dose was based on a previous study in rats [5]. The rats in the other two groups received an equal volume of 0.9% normal saline by oral gavage daily as a vehicle control. Food intake was recorded daily, and body weight was recorded every four days. During the experiment, three rats died from giant fibroadenomas involving the upper limb and chest (one case in each group), two rats died from intestinal obstruction (one in the CON group and one in the EBE group), and two rats died because of chronic infection (one in the AGE group and one in the EBE group) diagnosed by veterinarians. Following these deaths and exclusions, 6, 6, and 5 rats remained in the CON, AGE, and EBE groups, respectively. All the remaining rats were treated for 16 months, anesthetized with isoflurane (5% *v*/*v*) and euthanized by exsanguination via the descending aorta. The dorsal skin was shaved and treated with hair removal cream, and tissues were used to clean the remaining chemicals of the hair removal cream from the skin. All experiments were performed by investigators blinded to treatment group allocation.

### 2.5. Targeted AGEs Metabolites Measurement

UHPLC-MS was used to detect the concentrations of four types of AGEs in food, serum, and skin tissue. We measured the AGE content in a 25 μL liquid or 25 mg solid sample. Internal standards were used for accurate quantification. Briefly, free AGEs were extracted for instrumental analysis. For bound types of AGEs, extra hydrolysis and evaporation procedures were required. All surviving rats were included in the analysis.

### 2.6. Lipidomics

UHPLC-MS was used for lipidomics analysis. Orthogonal Projections to Latent Structures-Discriminant Analysis (OPLS-DA) was used to screen for the significance of differences. Different lipids (DLs) were enriched with a two-tailed hypothesis on the Lipidontology (LION) website [18]. In the subclass analysis, the Levene test was used to check the homogeneity of variance among the three groups. Subclasses with homogeneity of variance were analyzed with ANOVA and the Tukey HSD test for multiple testing correction; otherwise, Welch’s ANOVA and the Games-Howell test were used for differential analysis. All surviving rats were included in the analysis.

### 2.7. Statistical Analysis for Animal Study

A three-group analysis was conducted using one-way ANOVA or two-way ANOVA, and a multiple comparison test was conducted using Tukey HSD. Correlation was assessed by Spearman correlation analysis. Bar and line plots were generated using GraphPad Prism 10. Other graphs for lipidomics analysis were generated using the ‘ggplot2’ R (version 4.3.1) package. *p* < 0.05 was regarded as the cutoff value for significance.

## 3. Results

### 3.1. Roasted High-AGE Diet Exposure Induced Systemic and Cutaneous AGE Accumulation in Rats

Food intake and body weight were monitored throughout the experiment and summarized as monthly averages to assess the effects of the high-AGE diet and oral EBE intervention (Figure 1a,b), and no significant differences were observed in these two indicators. The levels of advanced glycation end products in the diet were quantified using UHPLC-MS/MS. After baking at 150 °C for 30 min, the contents of bound-CEL, bound-CML, free-CEL, and free-CML showed a significant increase in the diet (Figure 1c). To investigate AGE absorption in rats, we then detected serum AGE levels, as shown in Figure 1d, the above four AGEs all increased in the circulation. EBE supplementation significantly attenuated these increases. Similarly, bound-CEL and bound-CML increased after roasted high-AGE diet exposure in skin tissue, and these increases were attenuated by EBE treatment, whereas no significant between-group differences were observed in free AGE levels (Figure 1e).

### 3.2. Roasted High-AGE Diet Exposure Accelerated Skin Aging in Rats

The tissue samples were collected and stained with H&E to examine the histomorphology of the dorsal skin in rats (Figure 2a). Histological analysis revealed alterations in skin layer thickness. After 16 months of treatment, the stratum corneum of rats fed with a roasted diet exhibited marked thinning. However, EBE intervention ameliorated this thickness change. A parallel change was observed in the epidermis, likely due to the integral role of the stratum corneum. No significant differences were detected in the dermis among the three groups (Figure 2b). The relative fluorescence intensity of Ki67 showed a significantly reduced level in the AGE group compared to the other two groups, which indicated poor cellular proliferation (Figure 2c,d). These results indicated that roasted high-AGE diet exposure might affect the process of epidermal renewal, and EBE supplementation restored the epidermal turnover capacity. Staining for the classical senescence markers SA-β-Gal and p16 revealed increased senescence-associated staining after roasted high-AGE diet treatment. The EBE supplementation group showed a substantial reduction in senescent foci accumulation (Figure 2e,f). The senescent markers suggested the pro-aging effect of a roasted high-AGE diet and the anti-aging effect of EBE on the skin.

### 3.3. Roasted High-AGE Diet Exposure Induced Age-Related Molecular Changes in Rat Skin

Molecular phenotypes were detected to further investigate epidermal aging. Immunofluorescence and Western blotting were used to detect proteins in the epidermis (Figure 3a,b,e). K10 and K14 were significantly reduced in the AGE group, while they were increased by EBE intervention (Figure 3f). Quantitative assessment of K10 and K14 immunofluorescence explained the weakening in the stratum spinosum and stratum basale, respectively. Col17a1 expression was assessed in the basal epidermal layer and characterized keratinocyte differentiation capacity. Keratinocyte differentiation was disrupted in the AGE group and restored in the EBE group (Figure 3c,e,f). Roasted high-AGE diet treatment disrupted keratinocyte differentiation. EBE supplementation attenuated the alteration in dysfunction of epidermal basal cell differentiation. Western blotting again confirmed the variation in protein levels in the epidermis (Figure 3e,f). These results suggested that the roasted high-AGE diet impaired epidermal differentiation, thereby weakening epidermal layers; oral EBE intervention protected the differentiation capacity of the epidermis and the integrity of skin layers.

To further investigate the influences of roasted high-AGE diet and EBE supplementation on dermal aging, we investigated critical collagens and matrix metalloproteinases in the dermis. Sirius Red staining with polarization imaging separated type I and III collagen fibers into red and green differentially. A semi-quantitative analysis indicated that collagen I and the type I/III collagen ratio, but not type III collagen, were significantly reduced after roasted high-AGE diet treatment (Figure 3g,i). The ELISA quantification of type I collagen corroborated these alterations (Figure 3j). EBE supplementation slightly improved type I collagen but failed to reach significance. The upstream regulator collagenase Mmp-1 was determined by immunofluorescence and Western blotting and provided further evidence for collagen alteration (Figure 3h,k). Consumption of the roasted high-AGE diet was associated with reduced type I collagen content and increased MMP-1 expression. EBE supplementation reduced MMP-1 expression, whereas the increase in type I collagen content did not reach statistical significance.

### 3.4. Roasted High-AGE Diet Exposure Induced Skin Barrier-Related Molecule Disruption in Rats

To assess the potential effects of a roasted high-AGE diet and EBE supplementation on the skin barrier, stratum corneum proteins and tight junction proteins were quantified. Filaggrin and loricrin were measured as stratum corneum proteins and were significantly downregulated after the high-AGE diet (Figure 4a–c). EBE supplementation reversed the stratum corneum filaggrin levels, paralleling the epidermal thickness changes mentioned above. Similarly, Claudin-1 and Occludin, two tight junction proteins in skin tissue, showed a pattern similar to that of two stratum corneum proteins (Figure 4a–c). Furthermore, we examined the mRNA level of *AQP3*, a water channel protein, which was dramatically decreased by roasted high-AGE diet exposure and slightly restored by oral EBE supplementation, but failed to reach statistical significance; this may reflect water content or the water barrier in the skin (Figure 4c). These results suggest that the roasted high-AGE diet altered barrier-related molecules and that oral EBE supplementation could partially reverse the adverse effects on skin barrier-related molecules, but the protective effect on hydration-related molecules was limited.

### 3.5. Roasted High-AGE Diet Exposure Altered Skin Lipidomic Profiles in Rats

Given that lipids are the most abundant metabolites in the skin and are essential for epidermal homeostasis, we used UHPLC–MS/MS to characterize the effects of a roasted high-AGE diet and EBE supplementation on skin lipid profiles. A total of 1405 lipids were detected across all biopsies. Volcano plot analysis showed predominant lipid downregulation in the AGE group, with 162 decreased and only seven increased lipids, whereas EBE supplementation produced the opposite pattern, with 296 increased and six decreased lipids (Figure 5a), These results indicated marked lipid remodeling induced by the roasted high-AGE diet and a broad restorative effect of EBE.

We then focused on differentially expressed lipids (DLs). KEGG enrichment analysis showed significant enrichment of glycerophospholipid metabolism, glycerolipid metabolism, ether lipid metabolism, sphingolipid metabolism, and teichoic acid biosynthesis, suggesting alterations in energy storage, membrane composition, oxidative defense, and epidermal barrier function. LION enrichment further highlighted changes in lipid-related anti-inflammatory capacity, energy storage, and membrane biophysical properties, including intrinsic curvature, phase-transition temperature, headgroup charge, and bilayer thickness. The reduction in polyunsaturated lipid species suggested impaired antioxidant capacity and lipid storage after dietary AGE exposure, consistent with accelerated skin aging. In contrast, EBE supplementation enhanced lipid signatures associated with anti-inflammatory and antioxidant capacity and improved energy storage. Lipids with increased intrinsic curvature and negatively charged headgroups in the AGE group may reflect greater membrane flexibility and fluidity but reduced membrane stability. The accumulation of lysoglycerophospholipids also suggested membrane disruption, active secretion or endocytosis, and cellular stress or injury. Several changes, particularly those involving membrane lipids, were reversed by EBE (Figure 5b).

Among the 48 lipid subclasses, lysophosphatidylglycerol (LPG), lysophosphatidylserine (LPS), and Cer-AS were significantly altered by the roasted high-AGE diet and reversed by EBE, while eight additional subclasses were affected by either AGE exposure or EBE supplementation (Figure 5c and Figure S1b). Spearman correlation analysis was performed to determine whether lipid subclasses were associated with internal AGE exposure or skin AGE deposition (Figure 5d). Lipid alterations were more strongly related to serum AGEs, especially serum CEL, than to skin AGEs. Cer-AS, DG, and TG were negatively correlated with AGE levels, whereas several other lipid subclasses showed positive correlations. These findings suggest that AGEs may impair glycerolipid metabolism and sebum-related lipid storage. As key components of the stratum corneum, altered ceramides and glycerophospholipids also indicated barrier-related molecule disruption. Inflammatory lipid subclasses, including LPS and eicosanoids, accumulated in the AGE group and were reduced by EBE, supporting its inflammation-modulating effect.

Correlation analysis between lipid subclasses and skin phenotypes further showed that Cer-AS was mainly associated with barrier molecules, reinforcing its relevance to AGE-induced barrier impairment and EBE-mediated recovery (Figure 5e). LPG, DG, and TG showed broad correlations with multiple skin phenotypes, highlighting their potential contribution to skin aging.

Collectively, the roasted high-AGE diet induced lipid disturbances characterized by impaired energy storage, inflammation, oxidative stress, membrane instability, and barrier-related molecule disruption. EBE supplementation largely restored these lipid alterations, increasing DG and TG, reducing inflammatory and oxidative lipid signatures, and recovering barrier-associated Cer-AS. These lipid alterations were associated with the observed skin phenotypes, but their causal contribution to the potential protective effects of EBE remains to be established. Nevertheless, these findings support the role of a roasted high-AGE diet in aggravating chronological skin aging and suggest that EBE exerts protective anti-aging effects potentially through lipid metabolic remodeling.

### 3.6. Roasted High-AGE Diet Exposure Induced Activation of the JNK Signaling Pathway and Its Related Proinflammatory Response in the Skin

Based on the crucial role of the JNK signaling pathway and its related proinflammatory response in skin aging, we hypothesized the activation of Jnk and a related increase in inflammatory factors. Phosphorylated Jnk was upregulated in the roasted high-AGE diet exposure group and decreased in the EBE group, whereas total Jnk expression did not differ significantly among the groups, suggesting activated Jnk signaling (Figure 6a,b). For inflammatory assessment, qRT-PCR was used to detect Jnk-related inflammatory factors. The levels of *Tnf-α*, *Il-1β*, and *Il-12* were elevated after the high-AGE diet (Figure 6c). EBE supplementation substantially reversed the levels of *Tnf-α*, *Il-1β*, and *Il-6* in skin tissue. The difference in *Il-6* mRNA expression between the AGE and CON groups and the difference in *Il-12* mRNA expression between the EBE and AGE groups did not reach statistical significance. A roasted high-AGE diet induced inflammation, and the changes in phosphorylated Jnk and inflammatory cytokines were consistent with the involvement of Jnk-associated inflammatory signaling; however, a causal role of this pathway was not directly established.

## 4. Discussion

In this study, we investigated skin alterations associated with long-term consumption of a roasted high-AGE diet in rats and evaluated whether oral EBE supplementation attenuated these alterations.

AGE deposition in the skin is rarely reported in present studies. We quantified the exposure and deposition of four types of AGEs in in vivo skin research. These results confirmed the association between glycation and skin aging. We also found changes in epidermal and corneum thickness, which indicated that a roasted high-AGE diet impaired the proliferation, differentiation and keratinization of the epidermis [19,20,21]. Although no significant difference was observed in dermal thickness, the elevation of Mmp-1 and the subsequent collagen degradation explained the aging process in the dermis [22]. Our study demonstrated that long-term roasted high-AGE diet intake significantly increased skin AGE accumulation and was associated with more pronounced aging-associated changes in vivo.

EBE, which is derived from European bilberry, a plant belonging to the Vaccinium, is a group of phytochemicals rich in anthocyanins and anthocyanidins, and it can be digested and metabolized into a number of organic acids [23]. In vivo, EBE shows significant activity in reducing AGE levels in various organs [5]. Topical anthocyanin administration has been studied in repairing the skin barrier and attenuating the related inflammation and oxidative stress [24]. Due to the synergy of anthocyanins [25], natural plant extracts could be a better choice. EBE was also well tolerated, and the dose was based on previous human studies and finally decided as a five-fold body weight dose compared to humans [26]. As expected, the internal AGE exposure and skin AGE deposition were markedly decreased by EBE intervention, and our results in the meantime explained that EBE ameliorated skin aging by improving skin structural proteins and barrier-related molecules, although the improvements in type I collagen and *AQP3* did not reach statistical significance.

We tried to explain the mechanism from lipid aspects. Human trials have demonstrated that dietary AGE intake induces blood lipid disorders [12], and anthocyanins present a lipid-lowering effect [27]. Skin lipidomics revealed lower abundances of lipid species associated with energy storage in rats fed the high-AGE diet, which is in agreement with the current research status that lipids are lost with age [28]; the difference from other organs may be attributed to cell injury caused by energy consumption [29]. EBE intervention based on roasted high-AGE diet intake markedly restored energy storage, highlighting its anti-aging effect in the skin. We also found a reduction in Cer-AS indicating skin barrier regression, as Cer is a major component of the skin barrier [30,31]. Since our lipidomics data originated from full-thickness skin, future work is needed to confirm the exact skin anatomical location of the observed Cer-AS. The reduction in highly unsaturated fatty acids impairs the anti-inflammatory pathway, which may cause barrier dysfunction. Therefore we confirmed the activation of the JNK inflammatory pathway [32]. We also found membrane fluidity-associated lipid changes, and these might be one of the reasons for epidermal thinning and corneum peeling [33]. The observed lipid and Jnk-related changes may represent biological processes associated with the skin phenotypes, although their causal contributions require further confirmation.

A single-sex rat cohort was used in our study according to the previous skin natural aging research [34,35], because only female rats were included, the present study cannot assess sex-dependent responses or determine whether the findings are generalizable to male animals. Future studies including both sexes are warranted. Owing to the compound exposure, further studies should examine oral exposure to defined AGEs, and with the measurement of skin function indicators, the underlying mechanisms also need direct confirmation. Human trials are also needed for the validation of the protective effects of oral EBE supplementation on the skin.

In summary, we found that a roasted high-AGE diet accelerated skin aging in aged rats based on aging phenotypes and structural changes, mainly in the epidermal layer, and EBE reversed dermal structure and part of the barrier-related disruption. To fill the gap in existing research, we explored the chronic exposure to roasted high-AGE diet consumption, and also skin aging phenotypes potentially through lipid profile alteration and inflammatory signaling, influenced by a high AGEs diet.

## 5. Conclusions

Long-term consumption of a roasted high-AGE diet increased AGE accumulation in serum and skin and was associated with skin aging phenotypes and alterations in barrier-related molecules. Oral EBE supplementation attenuated several of these changes and partially restored skin lipid profiles. Changes in lipid metabolism and JNK-associated inflammatory signaling may contribute to these effects, although their causal roles remain to be established.

## Figures and Tables

**Figure 1:**
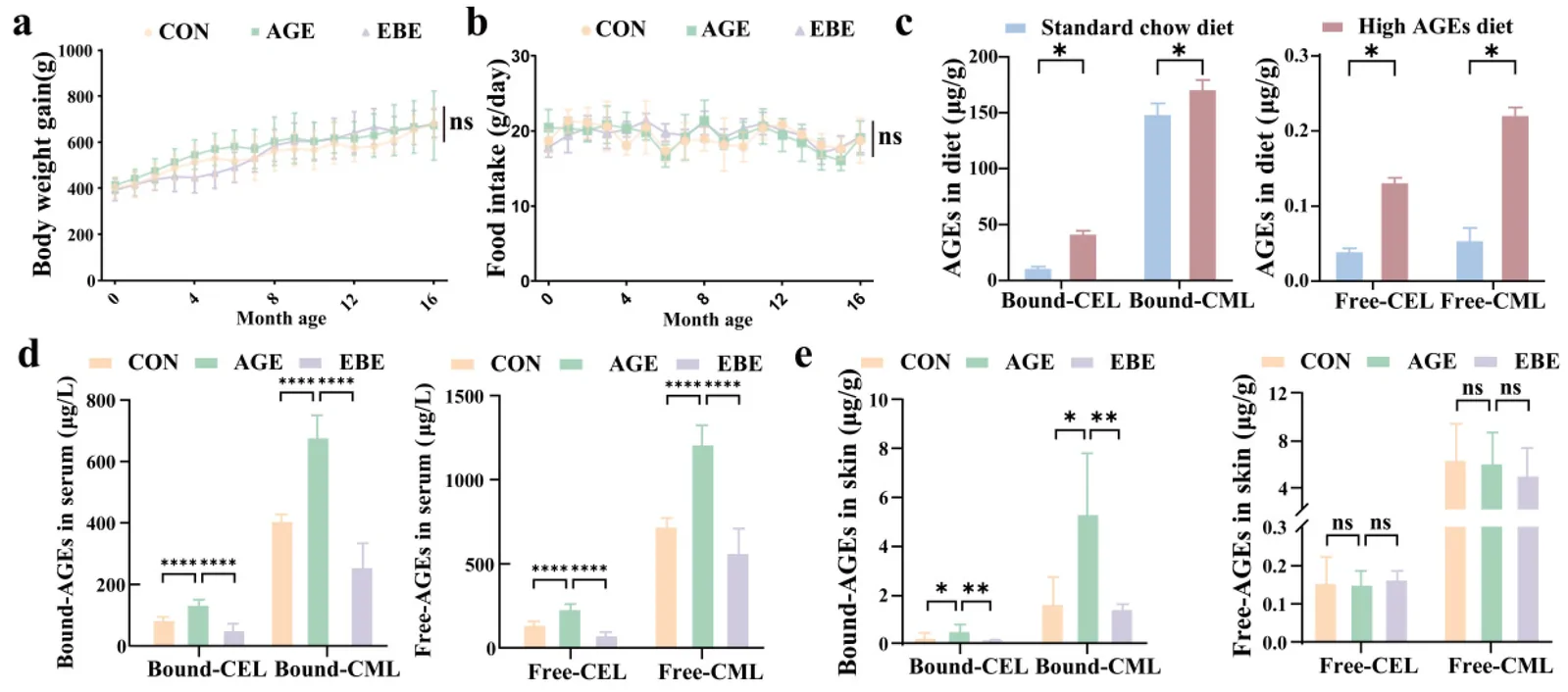
Advanced glycation end products (AGEs) levels of the roasted diet and effects of European bilberry extract (EBE) supplement on AGEs contents in rats. (**a**) Body weight throughout the experiment. (**b**) Daily food intake throughout the experiment. (**c**) AGEs levels in high-temperature roasted high-AGE diet. (**d**) The levels of bound-AGEs and free-AGEs in serum. (**e**) The levels of bound-AGEs and free-AGEs in skin tissue. AGEs levels in the roasted high-AGE diet were analyzed by *t*-test (**c**) while the three-group data were analyzed by two-way ANOVA (**a**,**b**) or one-way ANOVA (**d**,**e**), followed by Tukey’s multiple comparisons test (*n* = 5–6). * *p* < 0.05, ** *p* < 0.01, **** *p* < 0.0001, ns: non-significant.

**Figure 2:**
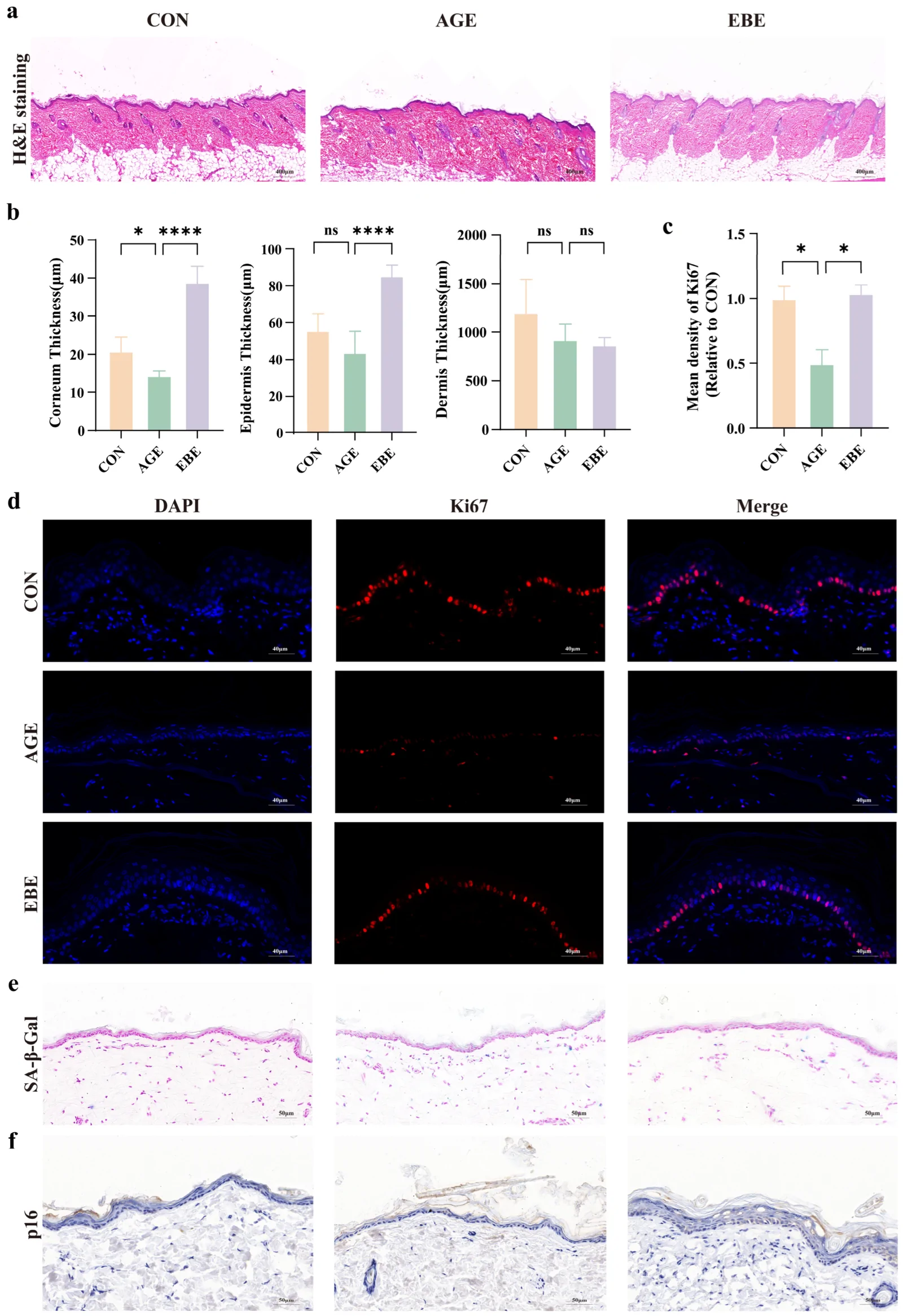
Effect of EBE supplementation on high AGEs-induced skin thickness and aging marker alterations. (**a**) Hematoxylin-eosin (HE) staining of the dorsal skin. (**b**) The thickness of different layers in skin tissue. (**c**,**d**) Ki67 levels in the epidermis (scale bar, 40 μm). (**e**) SA-β-Gal staining of dorsal skin (scale bar, 50 μm). (**f**) Immunohistochemistry for p16 of dorsal skin (scale bar, 50 μm). Data were analyzed by one-way ANOVA, followed by Tukey’s multiple comparisons test (*n* = 3). * *p* < 0.05, **** *p* < 0.0001, ns: non-significant.

**Figure 3:**
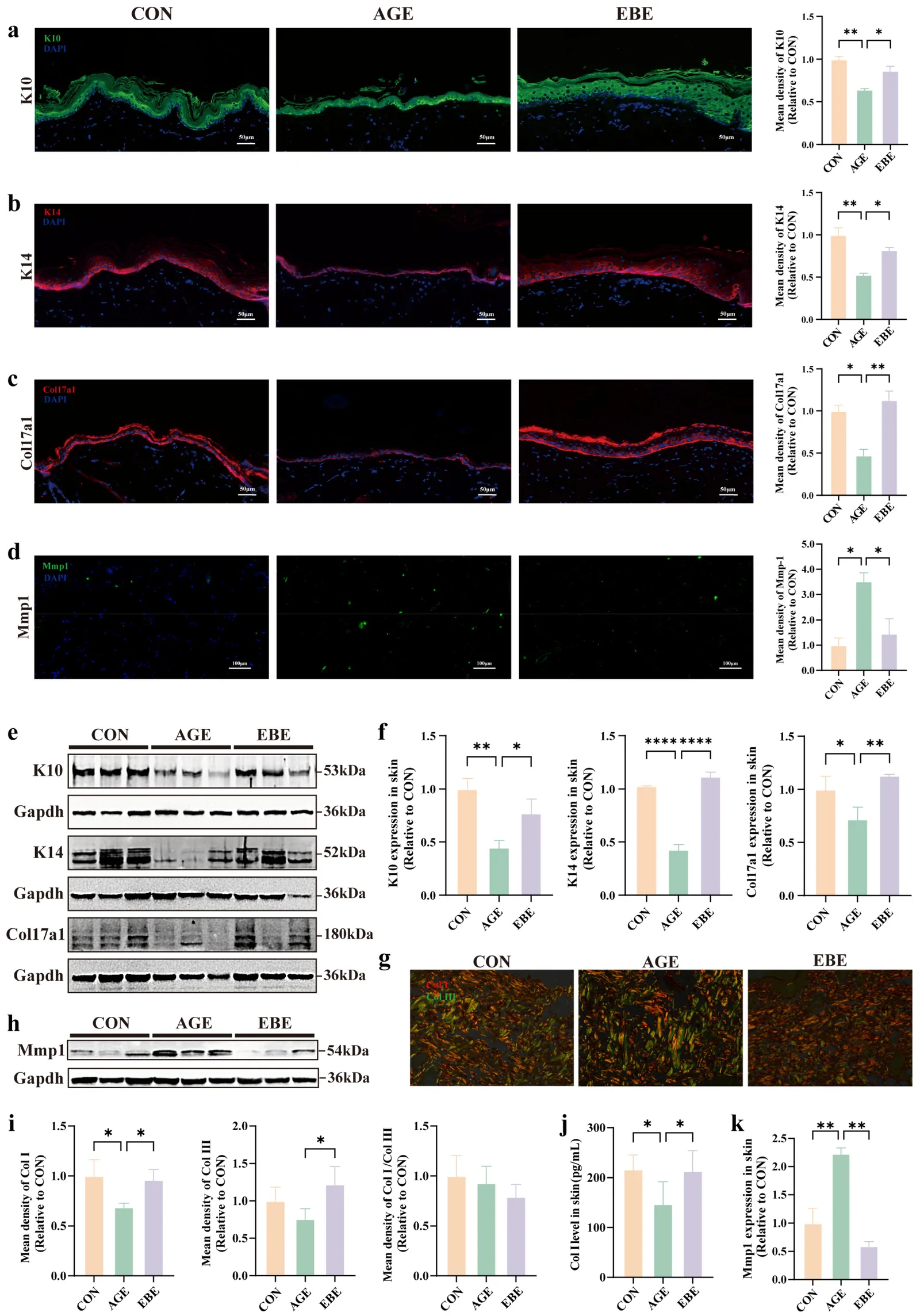
Effect of EBE supplementation on high AGEs-induced skin changes and quantification. (**a**) immunofluorescence for K10 in skin (scale bar, 50 μm). (**b**) Immunofluorescence for K14 in skin (scale bar, 50 μm). (**c**) Immunofluorescence for Col17a1 in skin (scale bar, 50 μm). (**d**) Immunofluorescence for Mmp-1 in skin (scale bar, 100 μm). (**e**,**f**) Western blot and quantified epidermal proteins. (**g**) Masson staining and polarized light microscopy for dermis collagen; type I collagen was shown in red, and type III collagen was shown in green (magnification: 200×). (**h**) Western blot of Mmp-1 in skin. (**i**) Semi-quantitative analysis of collagens in pathological sections. (**j**) Quantification of type I collagen detected by ELISA. (**k**) Quantified Mmp-1 expression in skin by Western blot. Data were analyzed by one-way ANOVA, followed by Tukey’s multiple comparisons test (*n* = 3). * *p* < 0.05, ** *p* < 0.01, **** *p* < 0.0001.

**Figure 4:**
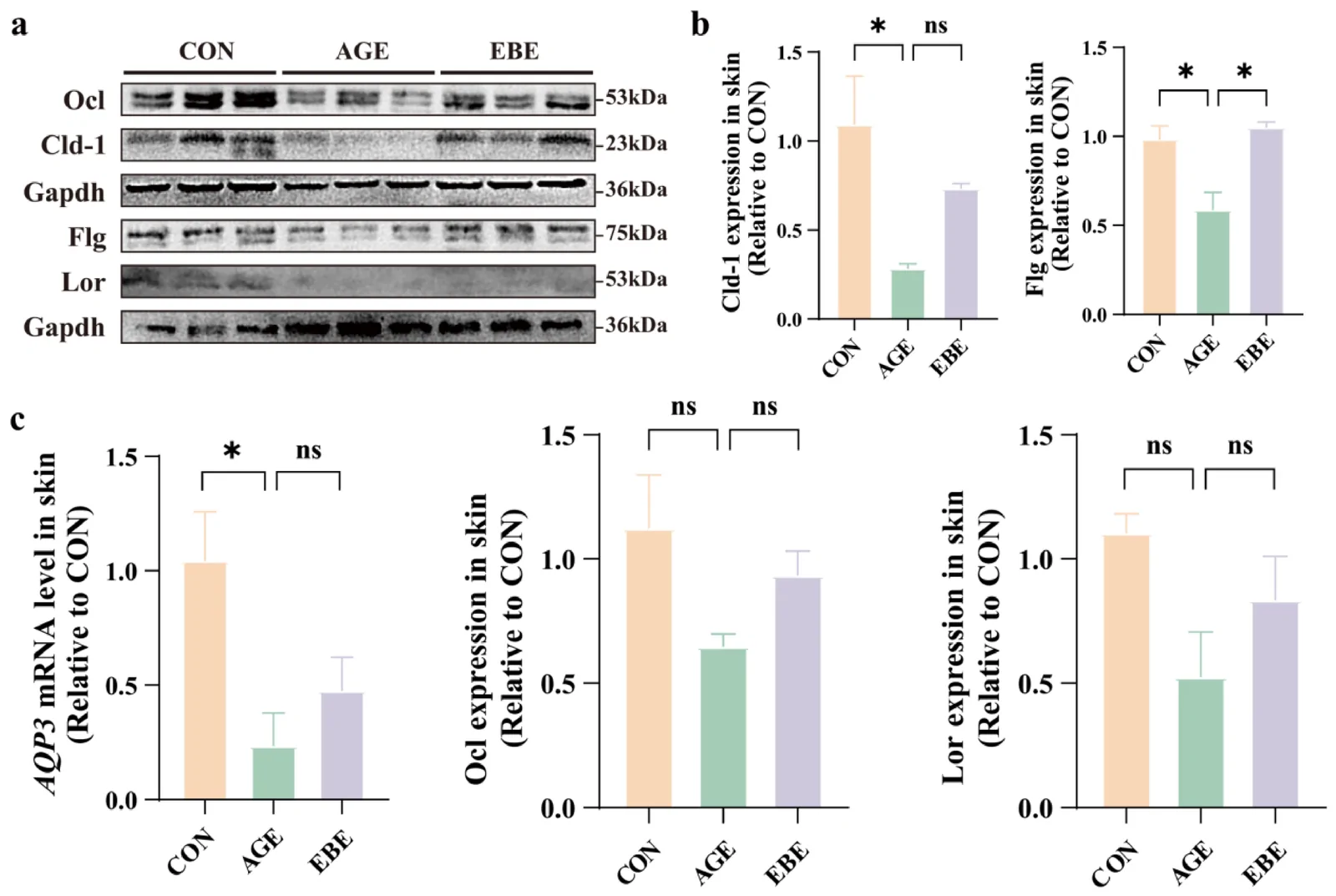
EBE supplementation alleviates high AGEs-induced barrier-related molecule disruption. (**a**) Western blot barrier of related molecules in skin. (**b**) Two barrier proteins were significantly reduced in the AGE group. (**c**) Skin hydration-related *AQP3* and the two other barrier-related protein expressions in skin. Data were analyzed by one-way ANOVA, followed by Tukey’s multiple comparisons test (*n* = 3). * *p* < 0.05, ns: non-significant.

**Figure 5:**
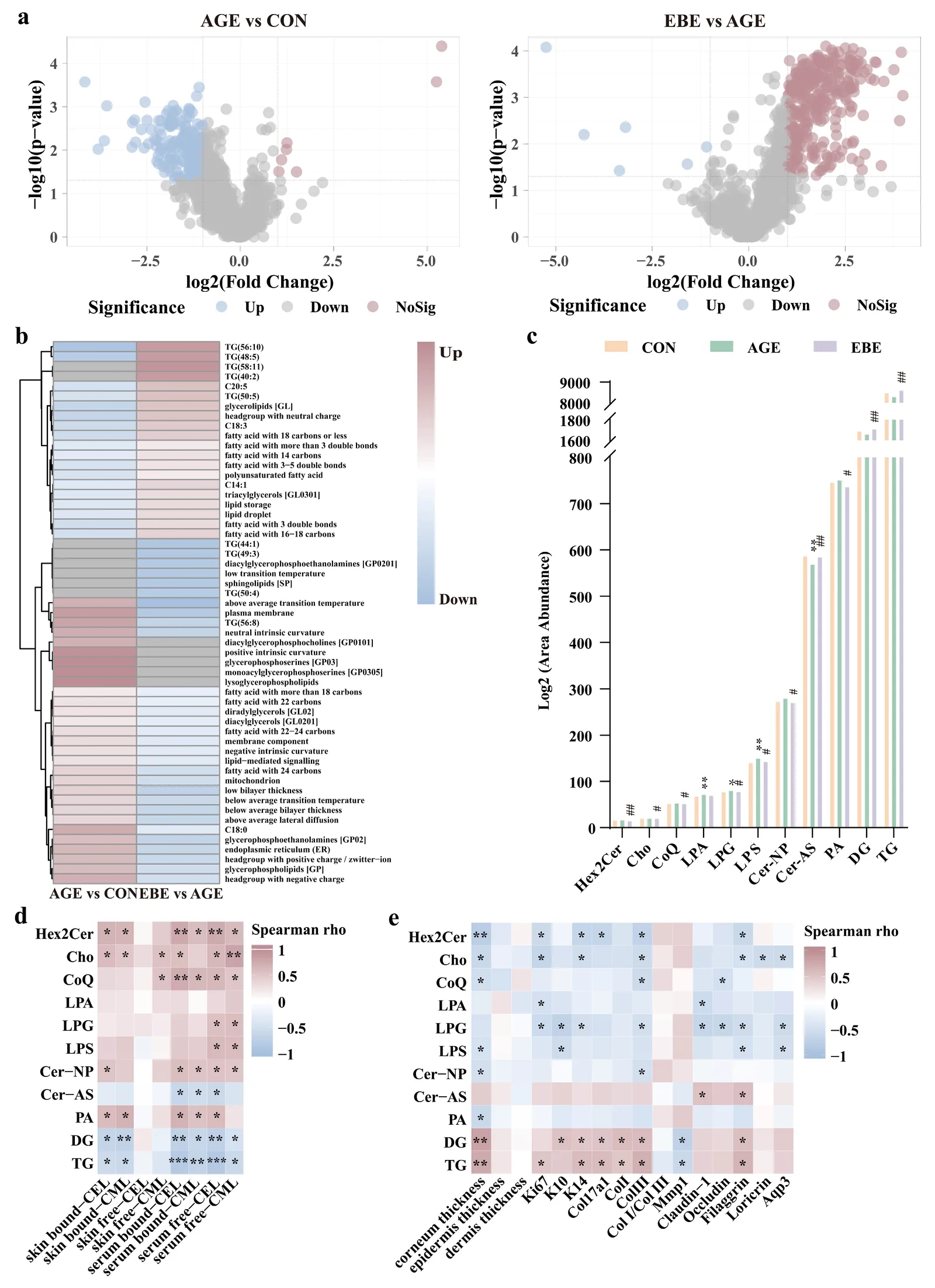
Lipidomics analysis of skin tissue exposed to roasted high-AGE diet and treated with EBE supplementation. (**a**) Volcano plot for the two comparison groups. (**b**) LION enrichment of Differential Lipids (DLs) in the two comparison groups. (**c**) Subclass lipid content in three groups. * AGE vs. CON, # EBE vs. AGE. (**d**) The correlation between AGEs levels and significantly altered lipid subclasses. (**e**) The correlation between skin aging phenotypes and significantly altered lipid subclasses. */# *p* < 0.05, **/## *p* < 0.01, *** *p* < 0.001.

**Figure 6:**
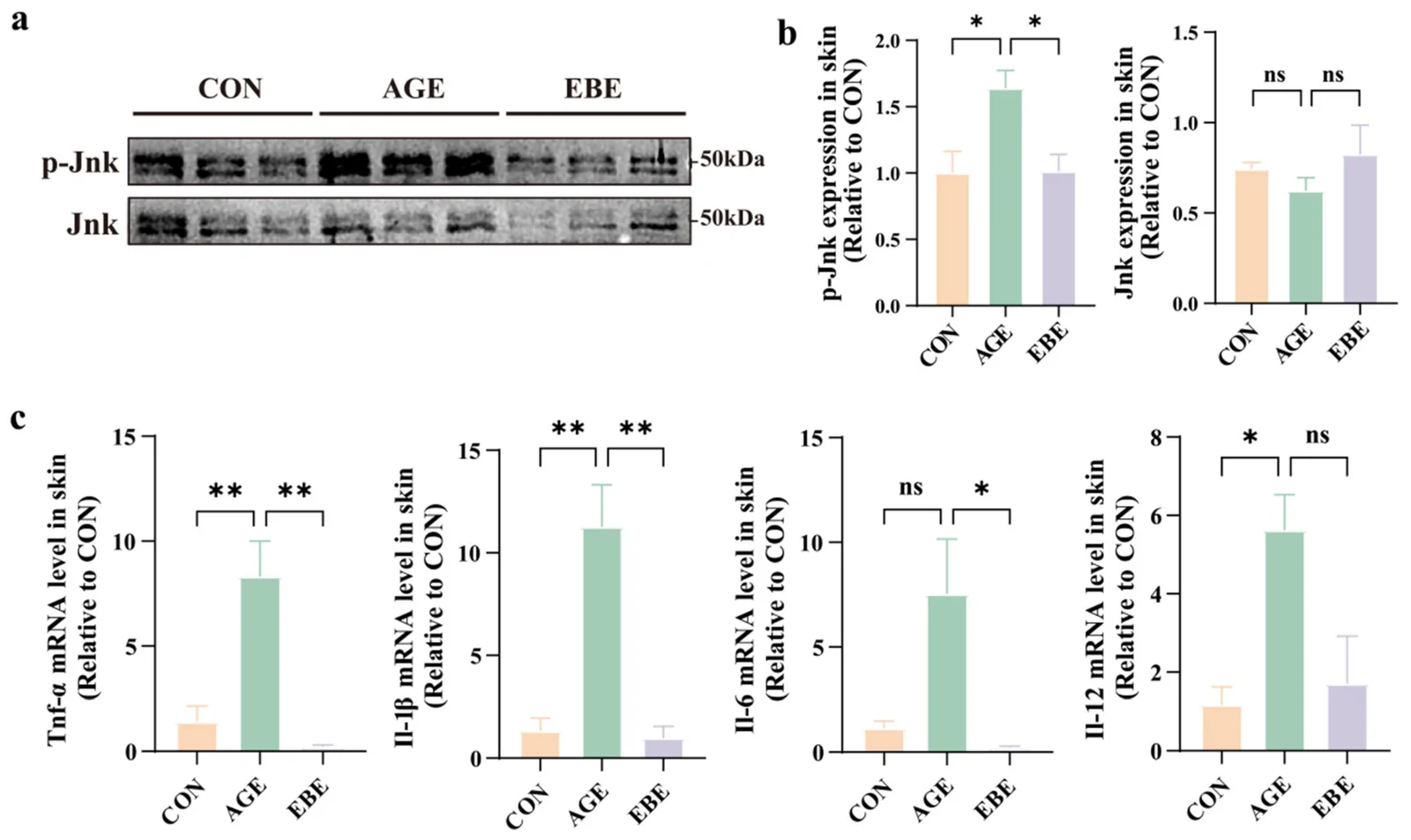
EBE supplementation ameliorates high AGEs-induced Jnk activation and related inflammation. (**a**,**b**) Western blot and quantified Jnk and phosphorylated Jnk in skin. (**c**) mRNA levels of Jnk-related inflammation factors in skin. Data were analyzed by one-way ANOVA, followed by Tukey’s multiple comparisons test (*n* = 3). * *p* < 0.05, ** *p* < 0.01, ns: non-significant.

## Data Availability

The lipidomics data generated in this study are available from the corresponding author upon reasonable request.

## Supplementary Materials

The following supporting information can be downloaded at: https://www.mdpi.com/article/10.3390/nu18193195/s1, Figure S1: Lipidomics analysis of skin tissue exposed to dietary AGEs and treated with EBE supplementation. (a) All 48 subclasses lipid content in three groups. (b) KEGG enrichment of DLs in the two comparison groups. (c) PCA analysis of lipidomics in three groups. */# *p* < 0.05, **/## *p* < 0.01, *** *p* < 0.001; Table S1: Primers for qRT-PCR. References [5,18,36,37] are cited in the supplementary materials.

## Abbreviations

The following abbreviations are used in this manuscript:

AGEs	Advanced Glycation End Products
Cer	Ceramide
Cer-AS	α-Hydroxy Fatty Acid Sphingosine Ceramide
CEL	Nε-(Carboxyethyl)lysine
CML	Nε-(Carboxymethyl)lysine
Col I	Collagen Type I
CON	Control
DAPI	4′,6-Diamidino-2-Phenylindole
DG	Diglyceride
DLs	Differential Lipids
EBE	European Bilberry Extract
ECL	Enhanced Chemiluminescent
ELISA	Enzyme-Linked Immunosorbent Assay
GAPDH	Glyceraldehyde-3-Phosphate Dehydrogenase
H&E	Hematoxylin and Eosin
HRP	Horseradish Peroxidase
KEGG	Kyoto Encyclopedia of Genes and Genomes
LDL-C	Low-Density Lipoprotein Cholesterol
LION	Lipidontology
LPG	Lysophosphatidylglycerol
LPS	lysophosphatidylserine
TC	Total Cholesterol
TG	Triglyceride
OD	Optical Density
OPLS-DA	Orthogonal Projections to Latent Structures-Discriminant Analysis
PCA	Principal Component Analysis
PQN	Probabilistic Quotient Normalization
PUFAs	Polyunsaturated Fatty Acids
qRT-PCR	Quantitative Real-Time Polymerase Chain Reaction
SA-β-Gal	Senescence-Associated β-Galactosidase
SC	Stratum Corneum
SD	Sprague-Dawley
SPF	Specific Pathogen-Free
UPF	Ultra-processed food
UPLC-MS	Ultra-High Performance Liquid Chromatography-Mass Spectrometry
VIP	Variable Importance in Projection
