# Supplementary material for: Effects of European Bilberry Extract on Skin Aging Associated with Consumption of a High Temperature Roasted Diet in Rats
Source: Nutrients. 2026 Sep 27;18(19):3195. doi: 10.3390/nu18193195 (PMC13666844; doi:10.3390/nu18193195)
Supplement: Supplementary file 1 [file nutrients-18-03195-s001.zip › nutrients-4545274-supplementary.pdf]

Supplementary methods

Animals

Twelve-week-old female Sprague-Dawley (SD) rats were purchased from Beijing HFK bio-Technology Co., Ltd and reared in a specific pathogen-free (SPF) facility (22 ± 2 °C, 50 % ± 5 %, 12 h dark–12 h light cycle) with free access to irradiated food and water. The Institutional Animal Care and Use Committee at Tongji Medical College, Huazhong University of Science and Technology, approved the animal experiments (IACUC number:4831).

Preparation of EBE

Fresh European bilberries were soaked in 50%-70% ethanol for about 7 hours and centrifuged for crude extraction. The crude extracts were then refined by reduced pressure and centrifuged, and the supernatants were collected. Sequentially, the supernatants were operated on microporous absorption resin, and washed with water, 70%-95% ethanol, and mixed with sugary water to obtain the pure European bilberry extracts. The pure extracts were then concentrated in a vacuum, dried, ground, and maltodextrin excipients to standardized. The final products were dissolved by ultrasound.

High-performance liquid chromatography-tandem mass spectrometry was used to quantify fifteen anthocyanins, and five anthocyanidins were detected in the extracts. Chromatographic column conditions were: mobile phase A was formic acid–water (8.5:91.5, V/V); mobile phase B was acetonitrile-methanol-formic acid-water (225:22.5:8.5:41.5, V/V/V/V); the program was 93% A→75% A in the first 35 min; 75% A→35% A in 35-45 min; 35% A→0% A in 45-46 min; 100% B in 46-50 min; 0% A→93% A in 50-51 min; 93% A in 51-60 min. The proper contents of anthocyanins and anthocyanidins were available in the previous study of our team[1].

Targeted AGEs metabolites measurement

UHPLC-MS was used to detect the concentrations of four types of AGEs in food, serum, and skin tissue.

Briefly, for liquid samples, 25μL of serum was vortexed sufficiently with an equal amount of internal standard (1 ppm CML-d_2 and 0.5 ppm CEL-d_4) and methanol/acetonitrile (1:3, v/v). Supernatants, obtained by centrifuged, were then dried and mixed with 250μL 5 mmol/L 9-fluorenylacetic acid aqueous. The organic solution was collected to detect free types of AGE. For solid samples, 25mg of skin tissue and food were weighed and mixed with distilled water to homogenize. The homogenate was processed in the same with the liquid samples.

Bound types of AGEs in liquid samples, 25μL serum was mixed thoroughly with 20 volumes of sodium borohydride and stood at room temperature for 2h, then 2mL trichloroacetic acid was added to the tube. The sediment, after centrifuging, was mixed with 50μL internal standard (1ppm CML-d_2 and 0.5ppm CEL-d_4) and 500μL hydrochloric acid solution and heated at 110 °C for 18h after being concentrated by nitrogen blowing and dissolved in 500μL pure water. The solution was centrifuged, and the supernatant was mixed with nine fluorovaleric acid aqueous solution as mentioned above. For bound AGEs in solid samples, skin tissue and food were weighed for 25mg and homogenized in 250μL distilled water for the following extract process, as the same with the liquid sample.

The conditions of the chromatographic column were as follows: ZORBAX Eclipse Plus C18 (100 mm × 2.1 mm × 1.8 μm) column was used for UHPLC-MS, the loading conditions of chromatographic column were described as following: 5 mM nine fluorovaleric acid aqueous solution as mobile phase A,

and acetonitrile as mobile phase B, for free types of AGEs, 0–2 min, 96 % A; 2–5 min, 96 % A → 75 % A; 5–6 min, 75 % A → 40 % A; 6–7.5 min, 40 % A → 20 % A; 7.5–8.5 min, 20 % A → 60 % A; 8.5–9 min, 60 % A → 96 % A; 9–14 min, 96 % A as the elution process. For bound types of AGEs, 0–1 min, 96 % A; 1–4 min, 96 % A → 80 % A; 4–7 min, 80 % A → 60 % A; 7–8 min, 60 % A → 40 % A; 8–9 min, 40 % A →20 % A; 9–11 min, 20 % A; 11–11.5 min, 20 % A → 96 % A; 11.5–17.5 min, 96 % A as the elution process.

The mass spectrum conditions were: positive ion scanning mode and multi-reaction monitoring scanning mode for the electrospray ion source, 4kV spray voltage, 45 psi sheath gas flow rate, 11 psi auxiliary gas flow rate, and 350 ℃ ion source heating temperature. The remaining conditions and data access were shown as previously described[2].

Histopathological analysis

Histopathological analysis was performed to observe the morphology and foci location. Skin tissue was fixed in 4% paraformaldehyde solution and blocked with paraffin. The sections were sliced into 7-8μm with a dewaxing and rehydration process, and stained with hematoxylin and eosin (H&E) or Sirius red after dehydration. As for Senescence-Associated β-Galactosidase (SA-β-Gal) staining, the slides were fixed with paraformaldehyde (PFA) additionally before staining. Three visual fields were randomly selected and quantified by ImageJ software. All measurements were obtained from three biological replicates and validated in three independent experiments.

Immunofluorescence & Immunohistochemistry

Immunological staining was performed to observe the molecule localization and processed semi-quantification. Skin frozen sections were used for immunofluorescence and immunohistochemistry staining. After antigen retrieval, the slides were incubated with primary antibodies and secondary antibodies, respectively. The primary antibodies were: Ki67 (Cat#84432-1-RR, Proteintech, China), p16 (Cat#bs-0740R, Bioss, China), Mmp-1 (Cat#10371-2-AP, Proteintech, China), K10 (Cat#18343-1-AP , Proteintech, China), K14 (Cat#10143-1-AP, Proteintech, China), and Col17a1 (Cat#31190-1-AP, Proteintech, China). For immunofluorescence staining, the secondary antibodies were Alexa Fluor 488-labeled (Cat#ab150077, 1:400, Abcam) and Alexa Fluor 555-labeled (Cat#ab150078, 1:400, Abcam). Nuclear staining with DAPI (Cat#B0025, Powerful Biology, China) is also needed. The slides were kept in the dark and scanned as quickly as possible to avoid photobleaching. For immunohistochemistry staining, HRP-conjugated secondary antibody was used and visualized with DAB substrate. Three visual fields were randomly selected and quantified by ImageJ software. All measurements were obtained from three biological replicates and validated in three independent experiments.

Quantitative real-time Polymerase Chain Reaction

Quantitative real-time Polymerase Chain Reaction (qRT-PCR) was used to quantify the gene transcription levels. Total RNA from skin tissue was isolated by RNAprep Pure Tissue Kit (TIANGEN, China), and HiScript III All-in-one RT SuperMix Perfect for qPCR (Vazyme, China) was used for gDNA clearance and reverse transcription. cDNA for PCR was mixed with Taq Pro Universal SYBR qPCR Master Mix (Vazyme, China) and primers and quantified by QuantStudio™ 7 Flex (ThermoFisher, USA). GAPDH was used as a preference. $2^{-\Delta\Delta CT}$ value was used to calculate gene expression levels. The primer sequences are shown in Table S1. All measurements were obtained from three biological replicates and validated in three independent experiments.

ELISA

The level of type I collagen was measured by Rat Collagen Type I (Col I) ELISA Kit (Nanjingjiancheng, China) following the instructions. Briefly, skin tissue was homogenized in PBS and diluted to prepare the experimental sample. First, add the sample into the well with the Biotinylated Ab working solution and incubate 30min. Second, wash the wells with diluted washing solution 5 times. Third, add the HRP working solution into the wells and incubate for 30 minutes. Fourth, wash the wells with diluted washing solution 5 times. Fifth, add chromogenic solution into the wells and incubate for 10min from light. At last, add the stop solution and measure the optical density (OD) at 450 nm wavelength immediately. All measurements were obtained from three biological replicates and validated in three independent experiments.

Western blotting

Western blotting was used to quantify the skin aging protein and related signaling pathway activation. Skin tissue proteins were quantified by Western blot. First, skin tissue was mixed with RIPA (Beyotime, China) solution and homogenized. After centrifuging in a pre-cold instrument, the supernatant was used for BCA (Beyotime, China) total protein quantification. Protein solutions were then mixed with 1/5 volume of loading buffer and electrophoresis. The primary antibodies were: K10 (Cat#18343-1-AP, Proteintech, China), K14 (Cat#10143-1-AP, Proteintech, China), Col17a1 (Cat#31190-1-AP, Proteintech, China), Mmp-1 (Cat#10371-2-AP, Proteintech, China), Jnk (Cat#AF6318, Affinity, China), and p-Jnk (Cat#AF3318, Affinity, China). The secondary antibodies were anti-rabbit (Cat#7074, 1:10000, CST) and anti-mouse (Cat#7076, 1:10000, CST). SuperKine Enhanced Chemiluminescent (ECL) Detection Reagent (Cat#BMU102, Abbkine) was used to expose target proteins. All measurements were obtained from three biological replicates and validated in three independent experiments.

Lipidomics

In each sample, 20 mg of skin tissue was weighed for lipidome examination. After homogenization and centrifugation, 1mL methyl tert-butyl ether/methanol (3:1, v/v) was used to extract the internal standard. Add 200μL water and centrifuge in a pre-cold instrument to get the supernatant and concentrate. Then, add 200μL acetonitrile/isopropyl alcohol (1:1, v/v) and centrifuged, the supernatant was used for the following UHPLC-MS analysis.

The conditions of the chromatographic column were as follows: Thermo Accucore™ C30 (100 mm × 2.1 mm × 2.6 μm) column was used for UHPLC-MS, the loading conditions of chromatographic column were described as following: acetonitrile/water (60/40, v/v) as mobile phase A, and acetonitrile/isopropyl alcohol (10/90, v/v) as mobile phase B. The gradient elution process was: 0min, A/B (80:20, v/v), 2min, A/B (70:30, v/v), 4min, A/B (40:60, v/v), 9min, A/B (15:85, v/v), 14min, A/B (10:90, v/v), 15.5min, A/B (5:95, v/v), 17.3min, A/B (5:95, v/v), 17.5min, A/B (80:20, v/v), 20min, A/B (80:20, v/v).

Data processing was done by MultiQuant to perform peak integration and calibration. Data analysis was then performed in R. The area of peak integration was unit variance scaled to perform Principal Component Analysis (PCA) analysis. Log transformation, centering, and unit variance scaling were performed as data preprocessing. Orthogonal Projections to Latent Structures-Discriminant Analysis (OPLS-DA) was used to calculate Variable Importance in Projection (VIP) value in the 'ropls' package and 'limma' package to screen for the significance of differences. Different lipids (DLs) were defined as

VIP > 1 and p-value < 0.05. DLs in two comparison groups (CON group vs AGE group, AGE group vs EBE group) were descendingly ordered by log2FoldChange and enriched with a one-tailed hypothesis on the Lipidontology (LION) website[3,4].

For the subclasses analysis, peak integration values were processed with log transformation and Probabilistic Quotient Normalization (PQN) normalization, and summed up to identify total abundance in each subclass. The Levene test was used to check the homogeneity of variance in three groups. Subclasses with homogeneity of variance were analyzed with ANOVA and Tukey HSD for multiple testing correction; otherwise, Welch's ANOVA and Games-Howell test were used for differential analysis.

Supplementary figure

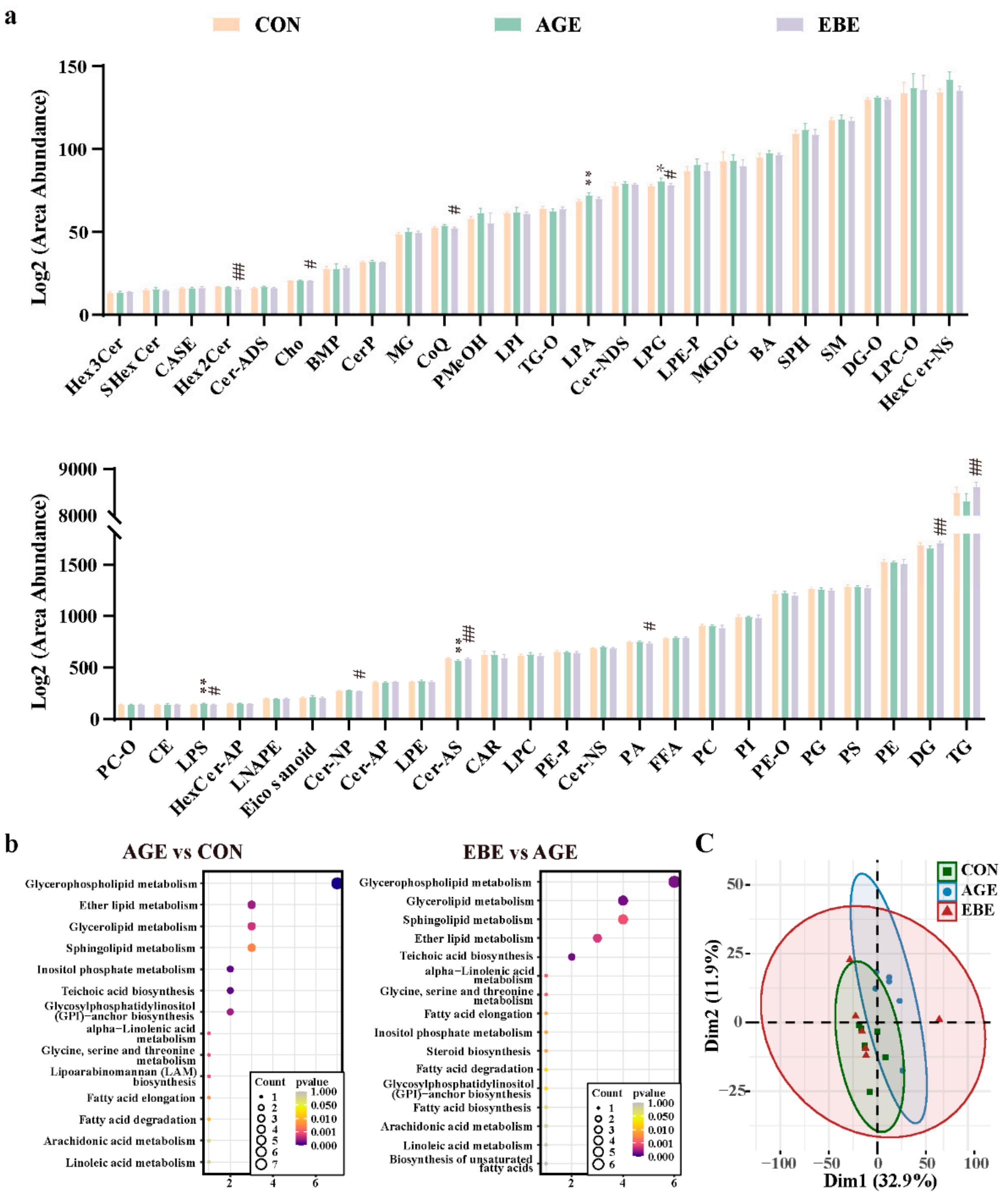

Figure S1 Lipidomics analysis of skin tissue exposed to dietary AGEs and treated with EBE supplementation. (a) All 48 subclasses lipid content in three groups. (b) KEGG enrichment of DLs in the two comparison groups. (c) PCA analysis of lipidomics in three groups. */#P < 0.05, **/##P < 0.01, *** P < 0.001.

Supplementary tables

Gene	Forward	Reverse
GAPDH	AACAGGGTGGTGGACCTCAT	GATGGTATTCGAGAGAAGGGAGG
IL-1β	TCCTCTGTGACTCGTGGGAT	GTTTGGGATCCACACTCTCCA
IL-6	CACTTCACAAGTCGGAGGCT	GAATTGCCATTGCACAACTCT
IL-12	ATCATCAAACCGGACCCACC	AGGAACGCACCTTTCTGGTT
TNF-α	ATGGGCTCCCTCTCATCAGTTCC	CTCCTCTGCTTGGTGGTTTGCTAC
Aqp3	AGATGCTCCACATCCGCTAC	AACCACAGCCGAACATCACA

Table S1 Primers for qRT-PCR

References

1. Mo, X.; Shen, L.; Wang, X.; Sun, Y.; Cheng, R.; Chen, W.; Chen, J.; He, R.; Liu, L. European bilberry extract reduces high-temperature baked food-induced accumulation of Nε-carboxymethyllysine and Nε-carboxyethyllysine in vivo. *Food Research International* **2024**, *197*, 115157, doi:10.1016/j.foodres.2024.115157.
2. Chen, L.; Wang, Q.; Lv, Y.; Xu, W.; Jiang, G.; Li, Y.; Luo, P.; He, R.; Liu, L. Association of plasma advanced glycation end-products and their soluble receptor with type 2 diabetes among Chinese adults. *Diabetes/metabolism research and reviews* **2024**, *40*, e3735, doi:10.1002/dmrr.3735.
3. Molenaar, M.R.; Jeucken, A.; Wassenaar, T.A.; van de Lest, C.H.A.; Brouwers, J.F.; Helms, J.B. LION/web: a web-based ontology enrichment tool for lipidomic data analysis. *GigaScience* **2019**, *8*, doi:10.1093/gigascience/giz061.
4. Molenaar, M.R.; Haaker, M.W.; Vaandrager, A.B.; Houweling, M.; Helms, J.B. Lipidomic profiling of rat hepatic stellate cells during activation reveals a two-stage process accompanied by increased levels of lysosomal lipids. *The Journal of biological chemistry* **2023**, *299*, 103042, doi:10.1016/j.jbc.2023.103042.
